# One-Pot Synthesized Amorphous Cobalt Sulfide With Enhanced Electrochemical Performance as Anodes for Lithium-Ion Batteries

**DOI:** 10.3389/fchem.2021.818255

**Published:** 2022-01-05

**Authors:** Long-Long Ren, Lin-Hui Wang, Yu-Feng Qin, Qiang Li

**Affiliations:** ^1^ College of Mechanical and Electronic Engineering, Shandong Agricultural University, Taian, China; ^2^ College of Information Science and Engineering, Shandong Agricultural University, Taian, China; ^3^ College of Physics, University-Industry Joint Center for Ocean Observation and Broadband Communication, Qingdao University, Qingdao, China

**Keywords:** amorphous, cobalt sulfide, one-pot synthesis, anodes, lithium-ion batteries, high electrochemical performance

## Abstract

In order to solve the poor cycle stability and the pulverization of cobalt sulfides electrodes, a series of amorphous and crystalline cobalt sulfides were prepared by one-pot solvothermal synthesis through controlling the reaction temperatures. Compared to the crystalline cobalt sulfide electrodes, the amorphous cobalt sulfide electrodes exhibited superior electrochemical performance. The high initial discharge and charge capacities of 2,132 mAh/g and 1,443 mAh/g at 200 mA/g were obtained. The reversible capacity was 1,245 mAh/g after 200 cycles, which is much higher than the theoretical capacity. The specific capability was 815 mAh/g at 800 mA/g and increased to 1,047 mAh/g when back to 100 mA/g, indicating the excellent rate capability. The outstanding electrochemical performance of the amorphous cobalt sulfide electrodes could result from the unique characteristics of more defects, isotropic nature, and the absence of grain boundaries for amorphous nanostructures, indicating the potential application of amorphous cobalt sulfide as anodes for lithium-ion batteries.

## Introduction

Lithium-ion batteries (LIBs) have been wildly used in small consumer electronics, electric vehicles, and medical apparatus as energy storage devices due to their advantages of high energy density, long cycle life, high working voltage, no memory effect, small self-discharge, and wide operating temperature range (Sun et al., 2010; Wang et al., 2020c; Gu et al., 2021; Li et al., 2021a; Li et al., 2021b; Li et al., 2021c; Li et al., 2021d; Zhao et al., 2021; Liang et al., 2022). However, to apply in large-scale energy storage projects and other high-power systems, the electrochemical properties of power density, rate capacity, cycle stability, and safety issue should be further improved (Sun et al., 2011; Zhang et al., 2019b; Wang et al., 2020b; Zhang et al., 2020a; Wang et al., 2021b). Current commercial graphite anode materials exhibit the advantages of high energy density, high conductivity, and security. Still, their low theoretical capacity of 372mAh/g and poor rate capability have confined the further development of LIBs(Zhang et al., 2019a; Hou et al., 2020; Li et al., 2020b; Gao et al., 2021; Wang et al., 2021a). Therefore, it is urgent to develop high-performance anode materials to meet the high power energy needs in the future (Zhao et al., 2019; Zhao et al., 2020; Liu et al., 2021a). It has long been discovered that cobalt sulfides (CoS, CoS_2_, Co_3_S_4_, Co_9_S_8_) have lithium storage ability and high reversible capacities (Yan et al., 2005; Shi et al., 2012; Gu et al., 2013). In order to achieve the practical application of cobalt sulfides as anode materials, the intrinsic drawbacks of low conductivity and significant volume expansion during cycles must be solved (Gu et al., 2013; Jiang et al., 2020). Many different crystalline nanostructures and morphologies have been designed and prepared to relieve the decomposition caused by the volume expansion. Carbon-based materials have also been introduced to increase the conductivity (Yan et al., 2005; Shi et al., 2012; Gu et al., 2013; Jiang et al., 2020). Yang *et al.* reported that the cubic phase of CoS_2_ was prepared by calcination at high temperature and exhibited the initial discharge capacity of 1,280 mA h/g and the reversible capacity of 350 mA h/g after ten cycles at 50 mA/g (Yan et al., 2005). Yan *et al.* prepared carbon-coated Co_9_S_8_ nano-dandelions by a facile solvothermal method, and the high reversible capacity of 520 mA h/g at the current density of 1 A/g (1.8 C) after the 50th cycle was obtained (Shi et al., 2012). Wang *et al.* prepared standard hexagonal CoS nanocomposites wrapped by graphene by a solvothermal method. The CoS nanocomposites exhibited a high reversible capacity of 749 mA h/g after 40 cycles at 62.5 mA/g (Gu et al., 2013). Wei *et al.* prepared the polycrystalline Co_9_S_8_/C composites by an electrospinning method, and the electrodes exhibited an initial discharge capacity of 823 mA h/g and a reversible capacity of 1,063 mA h/g after 200 cycles at 300 mA/g (Jiang et al., 2020). Even though good electrochemical performance has been observed in these crystalline cobalt sulfides, the poor cycle stability and the pulverization of the materials caused by the volume expansion still exist. Therefore, it is necessary to find a new way to solve these problems. Amorphous nanostructures always have more defects, which will provide more active sites. Furthermore, the isotropic nature and the absence of grain boundaries for amorphous nanostructures could improve the capacity to sustain high strain and the insertion of lithium ions, which is helpful to inhibit the volume expansion (Liu et al., 2013; Lu et al., 2018; Wu et al., 2019; Duan et al., 2021; Wu et al., 2021). Zhao *et al.* reported that the amorphous VO(PO_3_)_2_ exhibited a high initial discharge capacity of 1,297 mA h/g and a reversible capacity of 676 mA h/g after 150 cycles at the current of 100 mA/g, which is much higher than those of crystalline VO(PO_3_)_2_ due to the isotropic ions diffusion paths (Wu et al., 2021). Wu *et al.* reported that amorphous V_2_O_3_/C composite exhibited higher reversible capacity and superior cycling stability than crystalline V_2_O_3_/C composite, which accounted for the oxygen vacancies and amorphous phase (Wu et al., 2019). Yang *et al.* reported that compared to the crystalline Sn@C anodes, better rate capability, longer cycle life, and higher capacity had been observed for amorphous Sn@C anodes because of the defect sites and the improved strain regulation (Duan et al., 2021). However, as far as we know, the amorphous anode materials have not been systematically investigated, and the amorphous cobalt sulfides anode materials for LIBs have not been reported.

In this work, a series of amorphous and crystalline cobalt sulfide nanomaterials were prepared by a facile solvothermal method at different reaction temperatures. Due to the unique characteristics of more defects, isotropic nature, and the absence of grain boundaries for amorphous nanostructures, the amorphous cobalt sulfide exhibited superior electrochemical performance compared to the crystalline cobalt sulfide. The initial discharge and charge capacities of the amorphous samples are 2,132 mAh/g and 1,443 mAh/g, respectively, at 200 mA/g. The Coulombic efficiency sharply increased to 97.44% in the second cycle and maintained near 100% to the 200^th^ cycle. The high reversible capacity of 1,245 mAh/g after 200 cycles was observed. The specific capability was 815 mAh/g at 800 mA/g and increased to 1,047mAh/g when back to 100 mA/g, indicating the excellent rate capability. The amorphous cobalt sulfide nanomaterials with outstanding electrochemical performance have the potential application as anodes for LIBs.

## Experimental Section

### Materials and Batteries

**FIGURE 1:**
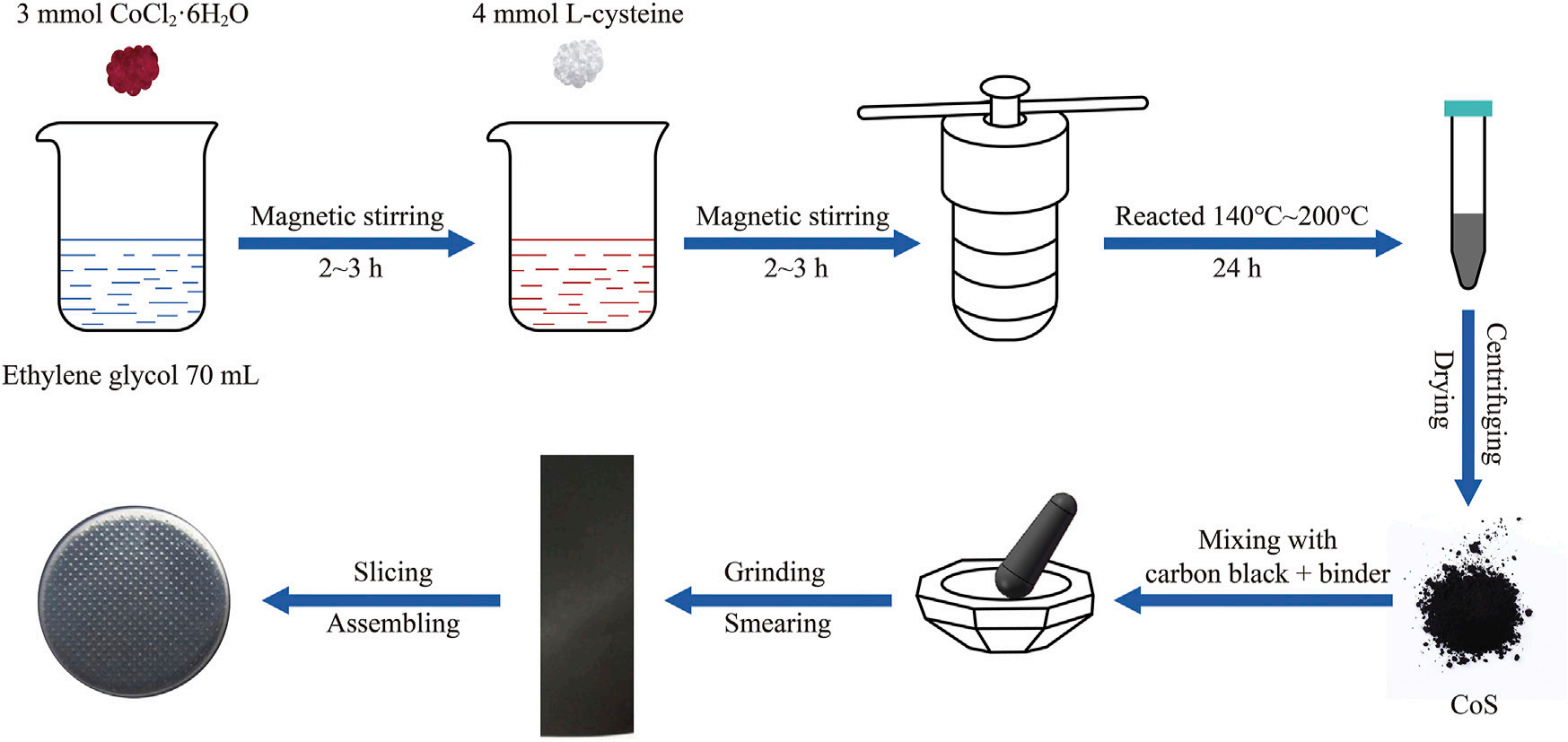
The schematic illustration of preparing the CoS materials and assembling half cells.

The schematic illustration of preparing CoS (amorphous and crystalline) materials and the assembling of the half cells (CR-2032) is shown in Figure 1. The cobalt sulfide nanomaterials were prepared as follows. 713.79 mg (3 mmol) of CoCl_2_·6H_2_O were added into 70 ml of ethylene glycol and magnetically stirred for 2∼3 h. 89.7 mg (4 mmol) of L-cysteine were added consequently and magnetically stirred for another 2∼3 h to dissolve completely. The mixed solution was divided into two Teflon-lined autoclaves (50 ml) and put in an air blast drying cabinet for 24 h at different reaction temperatures of 140°C, 160°C, 180°C, and 200°C, respectively. After the precipitates were alternately washed with deionized water and absolute alcohol several times, the precipitates were dried in a vacuum drying oven at 60°C for 12 h. Finally, the amorphous and crystalline cobalt sulfide nanomaterials were obtained. According to the reaction temperatures, the as-prepared cobalt sulfide nanomaterials were denoted by CoS-140, CoS-160, CoS-180, and CoS-200, respectively.

The cobalt sulfide nanomaterials, carbon black, and binder were mixed at a weight of 7: 2: 1 and roundly ground. The binder is carboxymethyl cellulose (CMC) dissolved in deionized water with a weight ratio of 10%. The black slurry was smeared evenly on the copper foil and then dried in a vacuum drying oven at 60°C for 12 h. The copper foil was punched into many disks with an area of 113 mm^2^. The average loading mass of the active materials is 0.82 mg/cm^2^. Finally, the half cells were assembled with the copper disks and the lithium metal foil in an argon-filled glove box. The diaphragm and electrolyte are the Celgard 2,250 film and 1M LiPF6 dissolved in a mixed solution of ethyl carbonate and dimethyl ethyl carbonate with a volume ratio of 1:1.

### Structure and Morphology

The structure was characterized by X-ray diffraction (XRD, Smart Lab, Rigku Japan) in the range of 20°–80° using a Cu Кα radiation. The morphology was further identified by a scanning electron microscope (SEM, GeminiSEM300, Zeiss, Germany).

### Electrochemical Performance Characterization

The electrochemical performance and impedance characteristics were measured by battery measuring systems (Land-ct2001A, China) and electrochemical workstation (CHI660E, China) in the potential range of 0.01–3.0 V at room temperature.

## Results and Discussion

### Structure and Morphology

**FIGURE 2:**
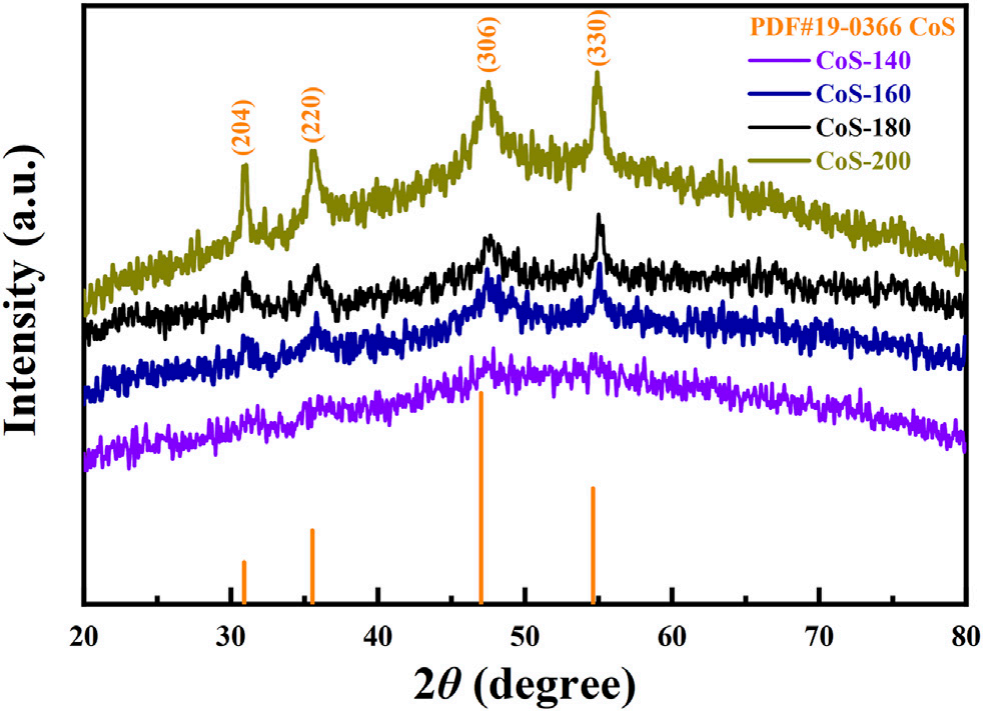
The XRD patterns of the CoS samples prepared at different temperatures.

The XRD patterns of the as-prepared materials reacted at different temperatures are shown in Figure 2. With the increase of the reaction temperatures, the diffraction peaks gradually become apparent. No diffraction peak is observed for the CoS-140 sample, indicating the amorphous or nanocrystalline structure due to low reaction temperature. The diffraction peaks gradually appear, and the intensities increase gradually for the CoS-160 and CoS-180 samples, which indicates a progressively crystallized process with the increase of the reaction temperatures. For the CoS-200 sample, the diffraction peaks are very remarkable, which means good crystallization. The diffraction peaks at 31.05°, 35.68°, 47.13°, and 54.91° are consistent with the standard card of PDF No. 19–0366 (CoS_1.097_), and these peaks correspond to the (204), (220), (306) and (330) crystal planes of hexagonal CoS_1.097_, respectively. In addition, no other diffraction peaks are observed, indicating the pure cobalt sulfide nanomaterials of our samples. The degree of crystallization increases with the reaction temperatures. A series of amorphous and crystalline cobalt sulfide nanomaterials were prepared by controlling the reaction temperatures.

**FIGURE 3:**
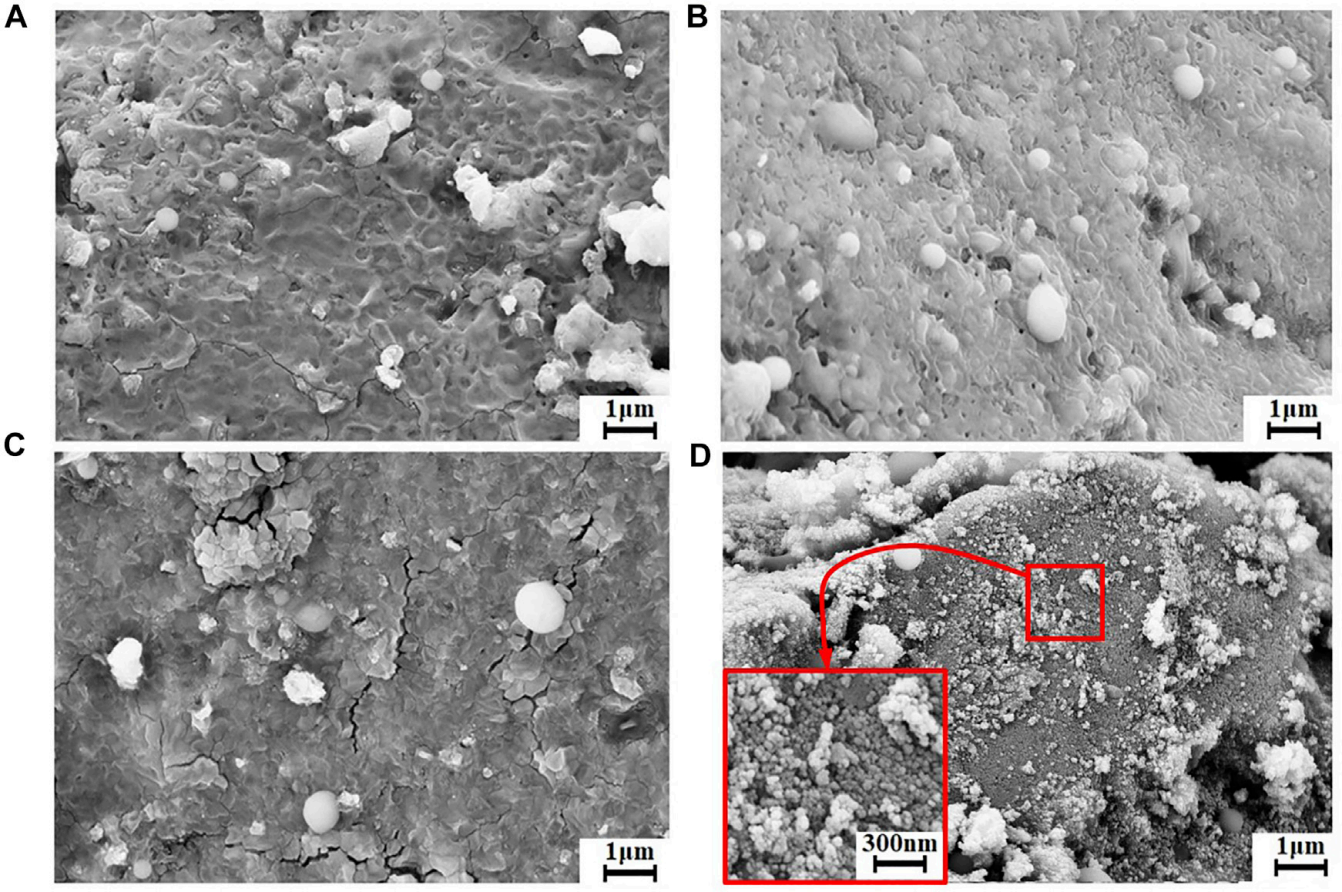
The SEM images of CoS-140 **(A)**, CoS-160 **(B)**, CoS-180 **(C)**, and CoS-200 **(D)**.

The morphologies of the amorphous and crystalline cobalt sulfide nanomaterials can be determined by the SEM images shown in Figure 3. From Figure 3A, the morphology of the amorphous CoS-140 sample is rough and irregular with many pits on the surface, and there are no noticeable regular crystalline grains observed, which is consistent with the absence of pronounced diffraction peaks shown in Figure 2. For the CoS-160 sample, some regular nanospheres are observed on the rough surface. And the regular nanospheres with different diameters should be crystalline structures. While for the CoS-180 sample, in addition to the regular nanospheres, some cracks are observed on the surface, which could result from the growth of the crystalline grains. A lot of small regular nanoparticles are observed for the CoS-200 sample, indicating crystalline growth of the sample, which strongly consists with the obvious diffraction peaks shown in Figure 2. Controlling reaction temperatures is crucial to synthesizing the cobalt sulfide nanomaterials with different amorphous or crystalline structures.

### Electrochemical Performance

**FIGURE 4:**
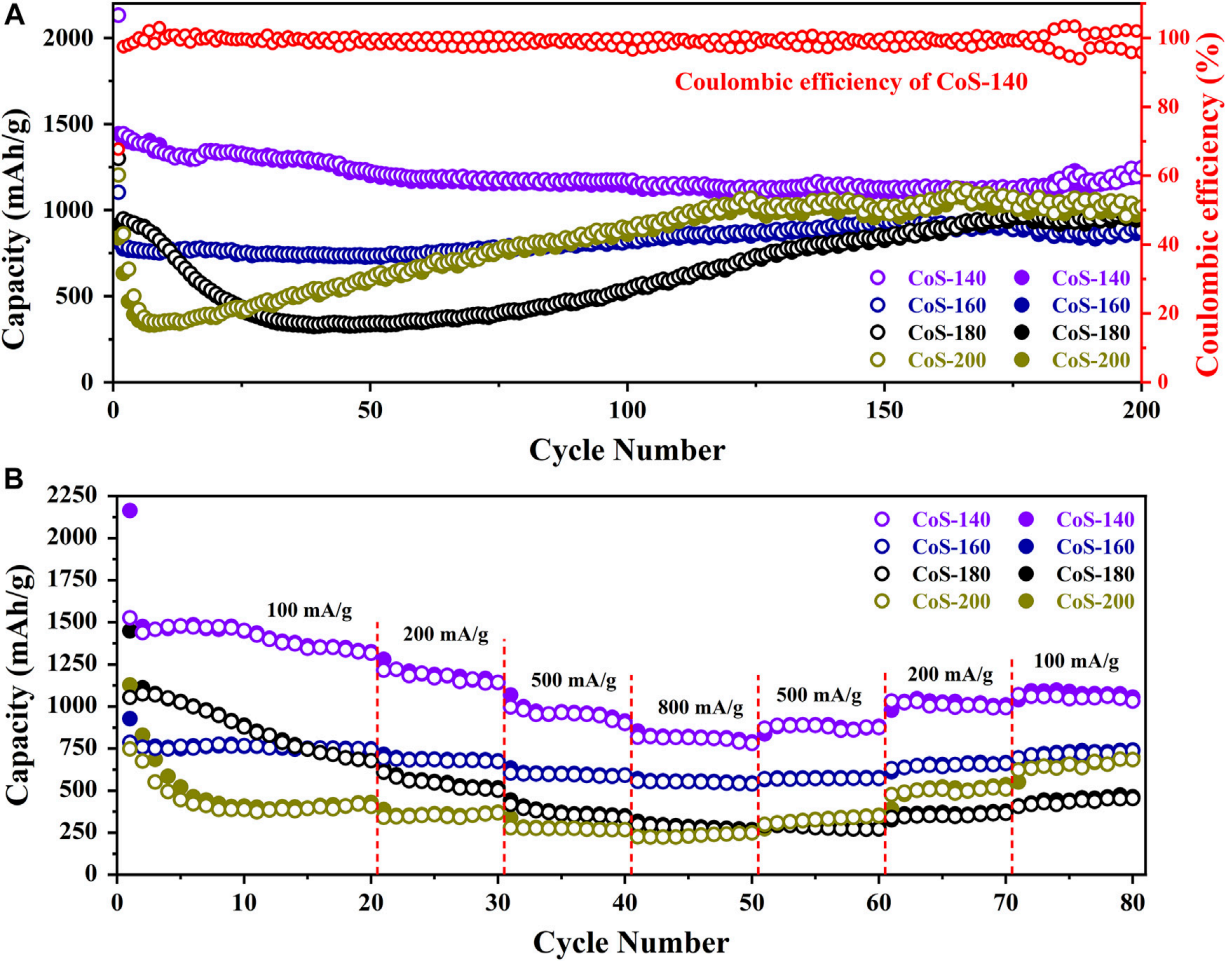
The cycle stabilities at 200 mA/g **(A)** and rate capabilities **(B)** of the CoS samples prepared at different temperatures. The solid and hollow circles represent the discharge and charge capacities, respectively.

In order to compare the electrochemical performance of the series of amorphous and crystalline cobalt sulfide nanomaterials, the cycle performance at 200 mA/g and the rate capability at different current densities were measured and shown in Figure 4. From Figure 4A, the samples of the CoS-140 and CoS-160 exhibit the relative constant reversible capacities, which indicates better cycle stability than that of the crystalline cobalt sulfide samples (CoS-160 and CoS-200). For the CoS-180 and CoS-200 samples, the specific capacities decrease first and then increase with the cycles, which is very common for the crystalline transition metal sulfides electrodes (Zhou et al., 2015). The first decrease could be due to the evolution of the SEI layers and the insufficient reaction of some active sites, and the subsequent increase could result from the polymeric gel-like layer and the decomposition of the crystal structure of CoS nanoparticles during the discharge-charge cycles (Zhou et al., 2020). However, the specific capacity of the CoS-200 sample decreases more sharply and obviously in the first cycles, which could result from the rapid decomposition of the crystalline structure during the cycles (Zhou et al., 2015; Wang et al., 2020b; Jiang et al., 2020). The CoS-140 sample exhibits the best cycling stability with the initial discharge and charge capacities of 2,132 mAh/g and 1,443 mAh/g, respectively. The Coulombic efficiency in the first cycle is 67.67% and radically increases to 97.44% in the second cycle, and maintains near 100% to the 200^th^ cycle. Significantly, the reversible capacity of 1,245 mAh/g after 200 cycles was obtained, which is much higher than the theoretical capacity of 589 mAh/g (Yan et al., 2005). The initial discharge capacity and the reversible capacity in this work and those of other reported cobalt sulfide-based electrodes are listed in Table 1, which indicates the outstanding electrochemical performance of the amorphous sample of CoS-140.

**TABLE 1:** The comparison of the electrochemical performance between this work and other reported cobalt sulfide-based electrodes.

Materials	Initial discharge capacity (mAh/g)	Reversible capacity (mAh/g)	Current density (mA/g)	References
Amorphous CoS	2,132	1,443 (200 cycles)	200	This work
Crystalline CoS	1,205	1,017 (200 cycles)		
C@Co_9_S_8_	848	520 (50 cycles)	1.8 C	Shi et al. (2012)
CoS_2_	1,280	350 (50 cycles)	50	Yan et al. (2005)
CoS/graphene	1,669	749 (40 cycles)	0.1 C	Gu et al. (2013)
Co_9_S_8_/C	2026	1,063 (200 cycles)	300	Jiang et al. (2020)
Co_9_S_8_-650@C	1,584	1,414 (100 cycles)	100	Zhou et al. (2015)
CoS_2_NP@G-CoS_2_	1,504	1,022 (50 cycles)	100	He et al. (2015)
CoS-NP/ACFs	1,137.3	576.7 (200cycles)	100	Yuan et al. (2020b)
CoS_2_-C/CNT	1,339	1,030 (120 cycles)	100	Ma et al. (2018)
Co_3_S_4_/CNF	991	742 (200 cycles)	1,000	Luo et al. (2019)
Si@C-Co_9_S_8_/C	1,441	1,399 (200 cycles)	100	Yuan et al. (2020a)
CMF@Co_9_S_8_-C	1,315	615 (450 cycles)	500	Zhang et al. (2020)
Co_4_S_3_/CNA@CC	1,200	720 (200 cycles)	1,000	Shi et al. (2020)
Co_9_S_8_	1,100	910 (100 cycles)	500	Lu et al. (2017)
CNTs@NC	1,366	914 (100 cycles)	100	Wang et al. (2020a)
Co_9_S_8_/Ni	1,580	720 (100 cycles)	100	Jin et al. (2016)
CoS_2_	1,542	737 (200 cycles)	1,000	Yu et al. (2016)
CoS_2_ NG	1,120	1,018 (50 cycles)	100	Qiu et al. (2015)
CoS_2_	1,416	883 (100 cycles)	100	Jin et al. (2015)

From Figure 4B, the samples of the CoS-140 and CoS-160 also exhibit better rate capability than that of the crystalline cobalt sulfide, which is consistent with the results of the cycle performance shown in Figure 4A. Even though the reversible capacity returns to 658 mA h/g when the current density goes back to 100 mA/g, the crystalline sample of CoS-200 exhibits the worst rate capability than other samples. The details of the average reversible capacities for the series of cobalt sulfide nanomaterials at different current densities are listed in Table 2, which also indicates the best rate capability of the amorphous sample of CoS-140 at each current density. The reversible capabilities of the amorphous CoS-140 sample are 1,450 mAh/g, 1,170 mAh/g, 958 mAh/g, and 815 mAh/g at 100 mA/g, 200 mA/g, 500 mA/g, and 800 mA/g, and the capabilities increase to 889 mAh/g, 1,015 mAh/g, and 1,047 mAh/g when back to 500 mA/g, 200 mA/g, and 100 mA/g, indicating the excellent rate capability. The outstanding electrochemical performance of the amorphous sample could result from the more active sites due to the more defects and the improved ability of the volume accommodation because of the isotropic nature and the absence of grain boundaries for the amorphous structure (Li et al., 2012; Liu et al., 2013; Wu et al., 2019; Wang et al., 2020b; Duan et al., 2021; Wu et al., 2021).

**TABLE 2:** The reversible capacities of the CoS samples at different current densities.

Samples	100 mA/g	200 mA/g	500 mA/g	800 mA/g	500 mA/g	200 mA/g	100 mA/g
CoS-140	1,451	1,170	958	815	889	1,015	1,047
CoS-160	766	684	595	551	572	643	723
CoS-180	879	546	361	279	280	347	434
CoS-200	389	357	276	230	326	485	658

**FIGURE 5:**
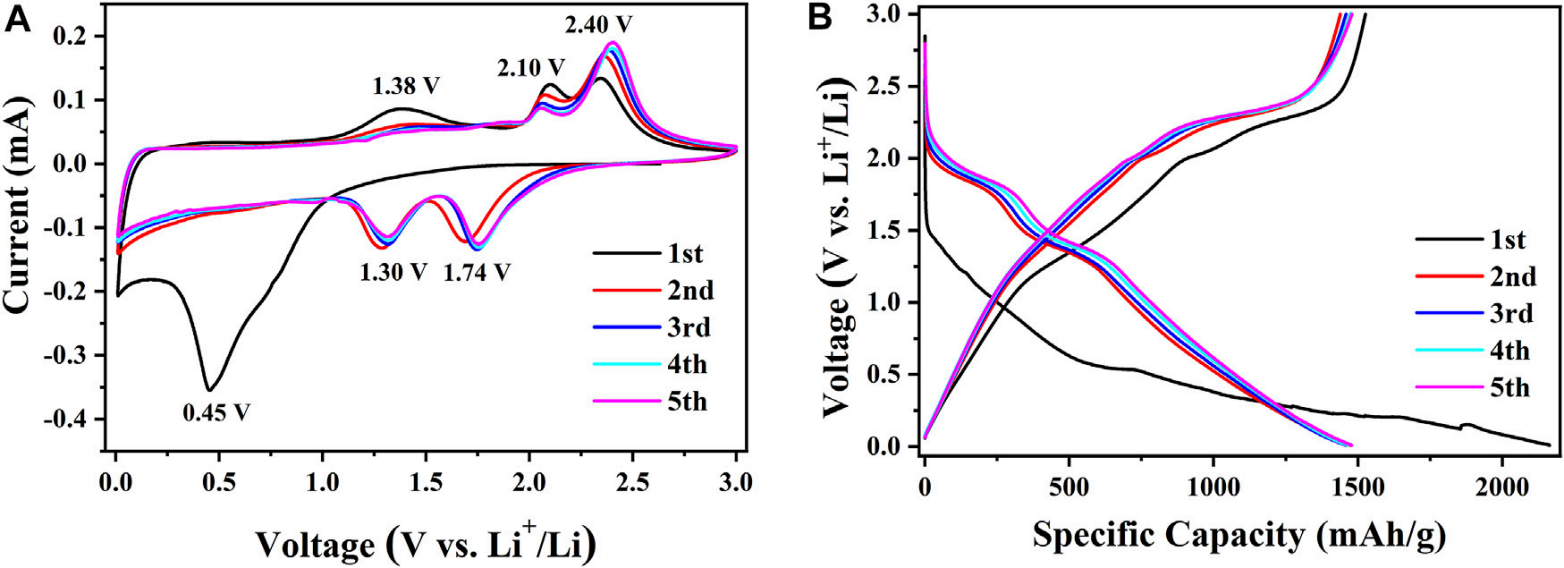
The first five CV curves at 0.1 m V/s **(A)** and the first five discharge-charge curves at 100 mA/g **(B)** for the amorphous CoS-140 sample.

Due to the best electrochemical performances, further investigation was focused on the amorphous CoS-140 sample. In order to comprehend the electrochemical reaction mechanism, the first five voltammetry (CV) curves of the amorphous CoS-140 sample were measured at a scan rate of 0.1 mV/s, as is shown in Figure 5A. In the first discharge process, there is a broad reduction peak at 0.45 V, which relates to the formation of the solid electrolyte interface (SEI) and the process of cobalt sulfide reduced to cobalt metal and Li_2_S (He et al., 2015; Zhou et al., 2015; Yuan et al., 2020b). In the following cathodic sweeps, the broad peak divides into two sharp peaks located at 1.74 and 1.30 V, which correspond to the formation of Li_*x*_CoS and the further convention reaction of Li_*x*_CoS to Co. metal (Ma et al., 2018; Yuan et al., 2020b). There is a broad peak at 1.38 V in the first charge process, which consists with the decomposition of the SEI layer (Zhou et al., 2015; Ma et al., 2018). This broad peak almost disappears in the following cycles due to the stability of the SEI layer, which is beneficial for the cycle stability (Zhou et al., 2015; Ma et al., 2018). There are also two oxidation peaks around 2.10 and 2.40 V in the five cathodic sweeps, which are consistent with the above reversible reactions of extraction of lithium ions to form CoS and the reduction process of Li_2_S to S (Zhou et al., 2015; Ma et al., 2018; Luo et al., 2019; Yuan et al., 2020b). The tiny changes of the positions for the two reduction peaks (1.74 and 1.30 V) and the two oxidation peaks (2.10 and 2.40 V) could result from a slight transformation of the structure (Zhou et al., 2015; Luo et al., 2019). The oxidation and reduction peaks almost coincide after the first cycle, indicating the stable electrochemical reaction process. The approximate overlap of the CV curves after the first cycle also shows excellent cycle stability and reversibility (Ma et al., 2018; Luo et al., 2019; Yuan et al., 2020b).

The first five discharge-charge curves at 100 mA/g are also shown in Figure 5B to compare the results of the CV curves. An extended voltage plateau from 0.63 to 0.15 V can be observed in the first discharge curves, which corresponds to the broad reduction peak at 0.45 V in the first CV cathodic sweep. There are two discharge plateaus around 2.10–1.70 V and 1.45–1.25 V in the following discharge curve, which consist with the division of the peak of 0.45 V into two peaks of 1.74 and 1.30V. For the first charge curve, there are three plateaus around 1.20 V-2.00 V, 2.00 V-2.20 V, and 2.20–2.46 V, corresponding to the three peaks of 1.36, 2.10, and 2.40 V in the first anodic sweep. The voltage plateau of 1.20–2.00 V disappears in the following cycles, indicating the stability of the SEI layer, which is in agreement with the disappearance of the oxidation peak at 1.38 V. After the initial cycle, the discharge-charge curves nearly overlap, which indicates the high reversible cycle stability and the reversible redox reactions of the amorphous sample.

**FIGURE 6:**
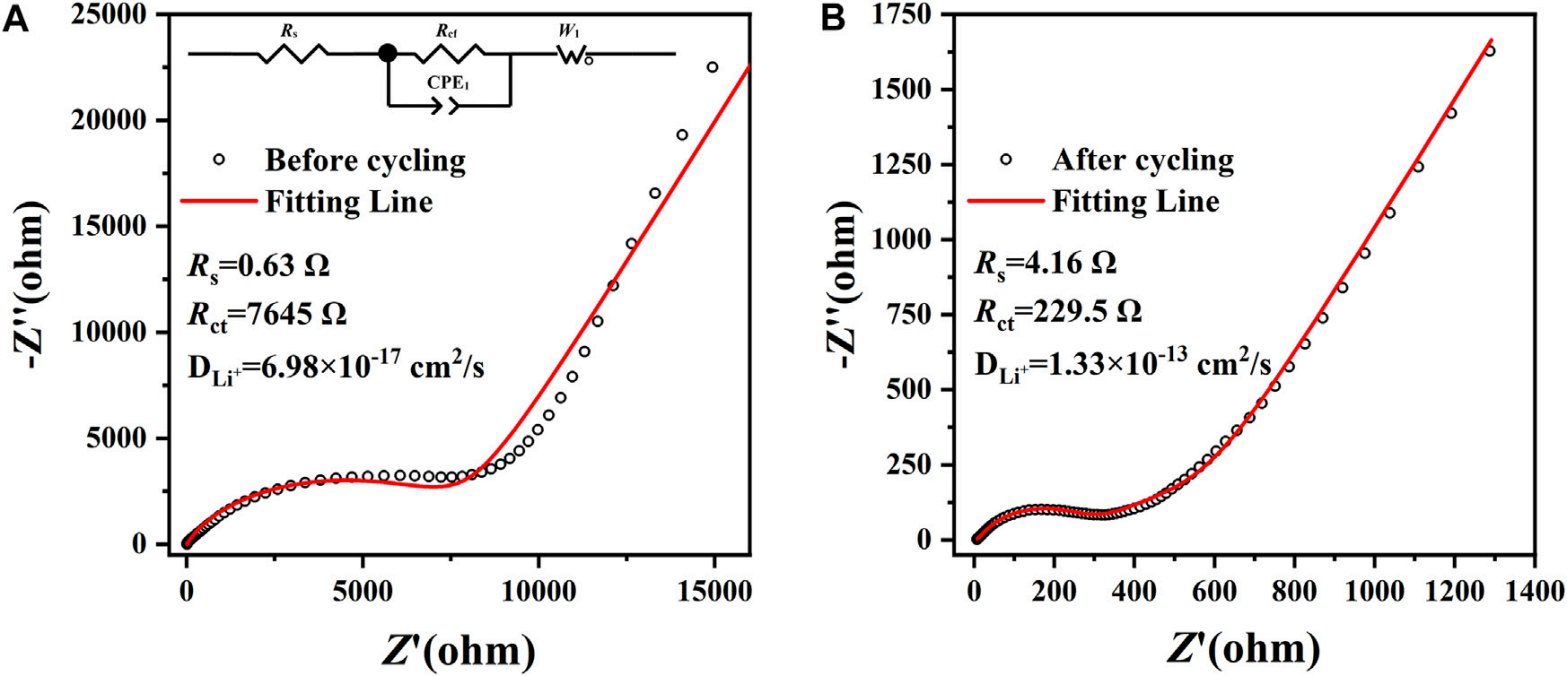
The EIS of the amorphous CoS-140 sample before **(A)** and after **(B)** cycling from 10^–2^ Hz–10^5^ Hz. The equivalent circuit is shown in the inset of **(A)**.

In order to deeply comprehend the enhanced electrochemical performance and the reaction kinetics of the amorphous CoS-140 sample, the electrochemical impedance spectroscopies (EIS) were measured from 10^–2^ Hz–10^5^ Hz before and after cycling, as is shown in Figure 6. Both the two Nyquist plots (black scatters) are composed of one depressed semicircle in high-frequency regions and one straight line in low-frequency regions, which can be well fitted by the equivalent circuit (red fitting lines) that is shown in the inset of Figure 6A. In the equivalent circuit, the parameter of *R*_s_ denotes the ohmic resistance of the electrode and electrolyte, and the parameter of *R*_ct_ signifies the charge transfer resistance (Wu et al., 2019; Duan et al., 2021; Wu et al., 2021). The fitted *R*_ct_ after cycling (229.5 Ω) is much lower than that of before cycling (7645 Ω), indicating the higher reaction kinetics activity during the cycles, which is in agreement with the excellent cycle stability and the rate capability shown in Figure 4. Moreover, the Li-ions diffusion coefficient (D_Li^+^_) can be obtained by the following equations (Wang et al., 2020b; Duan et al., 2021).

$$D_{Li^{+}}=\frac{R^{2}T^{2}}{2A^{2}n^{4}F^{4}C^{2}\sigma^{2}}$$ (1)

$$Z_{\mathrm{real}}=R_{s}+R_{ct}+\sigma \omega^{-1/2}$$ (2)

The parameters of *R*, *T*, *A*, *n*, *F*, *C*, *σ*, and *ω* are the general physical parameters gas constant, the measuring temperature, the surface area of the electrode, the number of transferred electrons, the Faraday constant, the concentration of lithium ions, the Warburg coefficient, and the angular frequency, respectively (Wang et al., 2020b; Yuan et al., 2020b). The value of *σ* could be fitted by Eq. 2 according to the EIS data in the low-frequency regions, and then the Li-ions diffusion coefficient can be calculated by Eq. 1. The Li-ions diffusion coefficient after cycling (1.33×10^–13^ cm^2^/s) is much higher than before cycling (6.98×10^–17^ cm^2^/s), which also indicates the better electrochemical kinetic activity during cycles. The three-dimensional isotropy structure of the amorphous CoS-140 could promote the penetration of the electrolyte and accelerate the diffusion velocity of the lithium ions into the active materials during the lithium storage process (Etacheri et al., 2015; Wu et al., 2018; Wu et al., 2020).

**FIGURE 7:**
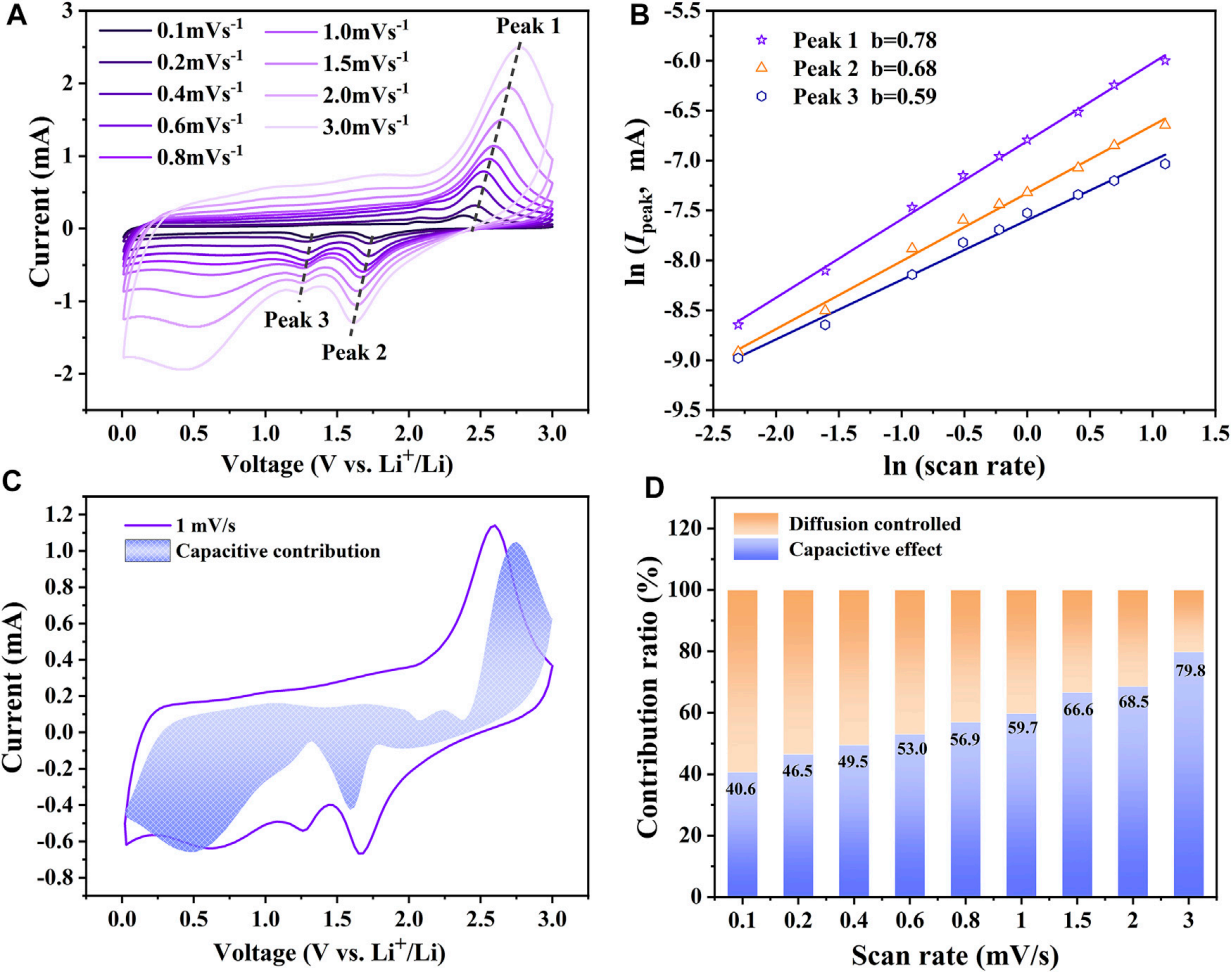
**(A)** The CV curves of the amorphous CoS-140 sample with different scan rates **(B)** The linear fitting of ln (*I*_Peak_) vs ln(*ν*). **(C)** Capacitive contribution at 1 mV/s. **(D)** Capacitive contributions at different scan rates.

It is necessary to further understand the reason for the fast reaction kinetic and the energy storage mechanism of the amorphous CoS-140 sample. The CV curves with different scan rates (0.1–3 mV/s) were measured and shown in Figure 7A. The CV curves maintain analogous shapes, while the areas enclosed by the CV curves and the identities of the redox reaction peaks gradually increase with the scan rates, which are always reported by other works of literature (Wu et al., 2019; Duan et al., 2021; Wu et al., 2021). The redox peaks are even evident at the high scan rate of 3 mV/s, indicating the high reaction dynamics (Wang et al., 2020d). The total energy storage of the electrode is generally contributed by two reaction processes of surface capacitive mechanism and diffusion mechanism (Duan et al., 2021), which can be roughly estimated by the following equations (Wang et al., 2020b; Yuan et al., 2020b).

$$I_{\text{Peak}}=av^{b}$$ (3)

$$1n\left(I_{Peak}\right)=b1\text{n}\left(v\right)+1\text{n}a$$ (4)

*I*_Peak_ represents the currents of the redox peaks at different scan rates marked by the arrows in Figure 7A, and *ν* denotes the corresponding scan rates. *a* and *b* are the variable parameters, and generally, the value of *b* is between 0.5 and one according to the different contribution ratios of the two parts. When *b* = 0.5, the electrochemical system is controlled by charge diffusion, while *b* = 1, the capacitive behavior is dominant (Wu et al., 2019; Wang et al., 2020b; Jiang et al., 2020; Duan et al., 2021; Wu et al., 2021). According to the linear fitting of ln (*I*_Peak_) vs ln(*ν*) shown in Figure 7B the values of *b* for the three redox reaction peaks are 0.68, 0.59, and 0.78, respectively, which indicates the mixed contribution of surface capacitive effect and diffusion-controlled process. The quantitative contribution of surface capacitive effect for the electrochemical system can be further analyzed by the following equations (Wu et al., 2019; Duan et al., 2021; Wu et al., 2021).

$$I=k_{1}v+k_{2}v^{0.5}$$ (5)

$$\frac{I}{v^{0.5}}=k_{1}v^{0.5}+k_{2}$$ (6)

*k*_1_ *v* and *k*_2_ *v* ^1/2^ represent the surface capacitive contribution and the charge diffusion contribution, respectively (Wu et al., 2019; Duan et al., 2021). A series of *k*_1_ and *k*_2_ can be obtained by the slope and intercept of *I*/ν^0.5^ vs *ν* ^0.5^ plots at different voltages. As shown in Figure 7C, the capacity contribution of the surface capacitive effect is 59.7% for the CV curves at the scan rate of 1 mV/s. The surface capacitive contributions for the capacities at different scan rates are shown in Figure 7D. The capacitive behavior contribution ratio gradually increases with the increase of the scan rates. The maximum contribution ratio of 79.8% is achieved at the scan rate of 3.0 mV/s, indicating the dominance of the capacitive behavior at a high scan rate, which is in agreement with the outstanding rate capability. The large contribution ratio of the capacitive behavior could result from the more surface defects and the inside void space of the amorphous structure for the CoS-140 sample, which is beneficial for the enhanced electrochemical performance (Lian et al., 2017; Li et al., 2020a; Liu et al., 2021b).

## Conclusion

In summary, through controlling the reaction temperatures, a series of amorphous and crystalline cobalt sulfide nanomaterials were prepared by a facile solvothermal method. Compared to the crystalline cobalt sulfide, the amorphous cobalt sulfide exhibited superior electrochemical performance with the initial discharge and charge capacities of 2,132 mAh/g and 1,443 mAh/g at 200 mA/g. The reversible capacity of 1,245 mAh/g after 200 cycles was obtained. After discharge-charge cycles at different current densities, the specific capability increased to 1,047 mAh/g when back to 100 mA/g. The outstanding electrochemical performance of the amorphous cobalt sulfide nanomaterials could result from the special structural characteristics of amorphous materials. The amorphous cobalt sulfide nanomaterials could be used as anodes for LIBs in the future.

## Data Availability

The raw data supporting the conclusion of this article will be made available by the authors, without undue reservation.
